# Progress in State Medicine

**Published:** 1897-04-17

**Authors:** 


### PROGRESS IN STATE MEDICINE.

At the moment of going to press, we are informed that, in a paragraph in our article of last week dealing with the septic tank system of dealing with sewage, we were in error in stating that on previous occasions the Local Government Board had withheld their sanction to the adoption of the process.

